# A Review of Metal Exposure and Its Effects on Bone Health

**DOI:** 10.1155/2018/4854152

**Published:** 2018-12-23

**Authors:** Juliana Rodríguez, Patricia Mónica Mandalunis

**Affiliations:** Department of Histology and Embryology, School of Dentistry, University of Buenos Aires, Argentina

## Abstract

The presence of metals in the environment is a matter of concern, since human activities are the major cause of pollution and metals can enter the food chain and bioaccumulate in hard and soft tissues/organs, which results in a long half-life of the metal in the body. Metal intoxication has a negative impact on human health and can alter different systems depending on metal type and concentration and duration of metal exposure. The present review focuses on the most common metals found in contaminated areas (cadmium, zinc, copper, nickel, mercury, chromium, lead, aluminum, titanium, and iron, as well as metalloid arsenic) and their effects on bone tissue. Both the lack and excess of these metals in the body can alter bone dynamics. Long term exposure and short exposure to high concentrations induce an imbalance in the bone remodeling process, altering both formation and resorption and leading to the development of different bone pathologies.

## 1. Introduction

Contamination with metals is a serious problem worldwide due to their toxicity and nonbiodegradability and their ability to accumulate in the environment and in living organisms.

Pollution of farmland soil and water is of great concern, since metal uptake by plants is a key route for the entry of metals into the food chain. The metals most frequently detected in the environment are cadmium, zinc, copper, arsenic, nickel, mercury, chromium, lead, and aluminum [1–3]. Most of these metals occur in nature as inorganic compounds. Some metals, such as titanium and iron, are potentially toxic to the body. Although arsenic is a metalloid it has been included in the present review since it is a widespread environmental toxic agent.

Cadmium occurs in the form of cadmium acetate, cadmium sulfate, cadmium chloride, cadmium oxide, and cadmium carbonate. Some fertilizers, cell phone batteries [4, 5], tobacco and tanning industry wastes, and even some metal platings used in jewelry contain this metal [6]. Hence, cadmium might be present in all types of foods. The half-life of cadmium in the body is approximately 10 to 30 years [7]. Cadmium intoxication can cause chronic renal failure [8], atherosclerosis and cardiovascular diseases [9], and cancer [10].

Zinc is an essential element and is found in nature in the form of zinc oxide and sphalerite (ZnS). It is used in the manufacture of batteries, steel, automobile parts, dyes, and cosmetics and as adjuvant in pesticides, fungicides, etc. [11]. Poisoning with zinc phosphide, a rodenticide, causes cardiovascular, respiratory, renal, and hepatobiliary failure, among other complications [12]. Exposure to zinc chloride, used as a smoke screen (military use), causes lung damage [13].

Copper, also an essential element found in all living beings, is involved in a number of biological processes, since it serves as a cofactor for several enzymes, mainly oxidases. Nevertheless, an increase in copper concentration in the body is toxic to cells. Copper is used as a fungicide, algaecide, and nutritional supplement, among other applications. Exposure to high levels of copper can result in liver and kidney damage, anemia, and immunotoxicity [14].

Nickel is used in more than 3000 metal alloys, batteries, and coinage and as a catalyst for several chemical reactions, in surgical and dental prostheses, etc. [15]. Effects of nickel exposure and intoxication include dermatitis, skin allergies, pulmonary fibrosis, and cardiovascular and kidney disease [16].

Mercury can occur as elemental mercury (Hg^0^) and inorganic mercury (Hg^0^, Hg^2+^, or mercury salts). Inhaled inorganic mercury is absorbed by the lungs and deposits in the brain, whereas ingested methylmercury is absorbed in the intestine and is deposited in several soft organs [17]. Organic mercury, which is bound to organic molecules, is used as a fungicide for seeds and grains, as well as in dental filling materials, preservative for vaccines, and fluorescent lamps [18]. Although all forms of mercury occur in all ecosystems, methylmercury is found in a larger proportion because it bioaccumulates in fish in contaminated areas through absorption and ingestion. Thus, the greatest source of methylmercury poisoning in humans is through ingestion of contaminated fish [19]. The half-life of mercury in the human body is approximately 70 days, after which 90% is excreted [20]. Mercury poisoning can cause cardiovascular disease, immunotoxicity [21], anemia, pulmonary fibrosis, Young's syndrome, renal failure, and hematoencephalic barrier damage, as well as endocrine disruption [22].

Chromium occurs in nature in different oxidation states, the most common being Cr (III) and Cr(VI). Chromium is widely distributed in the environment, in rocks, soil, water, dust, and volcanic ash. Chromium (III) in particular occurs naturally in some foods such as meat, eggs, fish, whole grain cereal, nuts, fruits, and vegetables and is essential to the human body in small doses due to its involvement in carbohydrate metabolism and regulation. Chromium (VI) is a waste product of industries including electroplating, leather tanning, and textile industry and of fossil-fuel combustion [23]. Chromium exposure causes dermatitis, allergies, as well as respiratory, gastrointestinal, neurologic [24], and reproductive problems [25], and cancer [26].

Lead is found in nature in different forms, such as ionic lead, lead salts, and tetraethyl lead or bound to organic compounds (organic lead) [27]. Major sources of environmental contamination include mining and steel, metal, and other industries. Lead is used in the manufacture of batteries, mainly car batteries, electrical systems, piping, construction materials, petrol, and sulfuric acid production, as a base for alloys and paints, as an antiknock agent for automotive gasoline, and for radiation shielding [28]. Lead poisoning can cause anemia [29], hypertension, risk for stroke and cardiovascular disease [30], and central and peripheral neurotoxicity [31].

Aluminum is a ubiquitous metal in the earth's crust. It is a trivalent metal (Al^3+^), and its behavior in aqueous solutions varies with pH (some of the compounds it can form include Al(OH)_2_^+^, AL(OH)^2+^, and Al (OH)_4_^−^ ) [32]. Aluminum is widely used to manufacture beverage cans and antacids, as a base for paints and cosmetics, and so forth. It is known that aluminum affects the hematopoietic and nervous systems and the skeleton, causing hemodialysis encephalopathy, anemia, aluminosis, osteomalacia, and osteoporosis, among other adverse health effects [33].

Titanium is a transition metal and is found in water, soil, and air. It is absorbed little by plants and animals. The routes of entry into the body are ingestion and inhalation during extraction and processing of the metal, which has been detected in the lungs, lymph nodes, brain, and blood [34]. Due to its resistance to corrosion and its mechanical properties, titanium is widely used in medicine for the manufacture of orthopedic prostheses and dental implants.

Iron is considered the second most abundant metal in nature and is essential to a number of biological processes. It catalyzes the formation of free radicals through the Haber-Weiss reaction, causing oxidative stress in different cells [35]. Genetic hemochromatosis is a disorder characterized by intestinal hyperabsorption of iron. The toxicity generated by the accumulation of iron in body tissues can cause cirrhosis, liver carcinoma, heart failure, diabetes mellitus, and osteoporosis [36].

Arsenic is not a metal but a metalloid that occurs as inorganic As (III) (arsenic oxide, sodium arsenite, etc.) and As (V) (arsenic pentoxide, arsenic acid, sodium arsenate, etc.) and as organic arsenic species [37]. Arsenic poisoning causes cancer and skin diseases (hyperpigmentation, hyperkeratosis), as well as liver, kidney, cardiovascular, respiratory, gastrointestinal, and nervous disorders, among other health effects [38].

Table 1 shows the levels below which the aforementioned metals cause no adverse effects.

### 1.1. Bone Tissue

Bone tissue is the main component of the skeletal system. It is made up of different cell types (osteoblasts, osteoclasts, osteocytes, and bone lining cells) and a calcified extracellular matrix. It is made up of minerals (50-70%), organic matter (20-40%), and water (5-10%). Around 90% of the organic component of the extracellular bone matrix is type I collagen, which is synthesized by osteoblasts and is deposited in layers in mature or lamellar bone [39].

The RANK/RANKL/OPG axis regulates osteoclast recruitment and activity. RANKL belongs to the TNF family and is expressed on the surface of osteoblasts. Upon binding to its receptor (RANK) on the surface of hematopoietic precursor cells, it induces osteoclast formation in the presence of macrophage-colony stimulating factor (MCS-F). Osteoprotegerin (OPG) is a glycoprotein belonging to the superfamily of TNF receptors, which by binding to RANKL prevents activation of RANK, inhibiting osteoclast formation [40].

Alkaline phosphatases are a family of enzymes that are important to the mineralization process. There are 4 isoenzymes in the human body: 3 are tissue-specific (intestinal, placenta, and germ cells) and one is nonspecific and is expressed in high levels in bone, liver, and kidney. The nonspecific isoenzyme is expressed in hypertrophic chondrocytes and osteoblasts. Alkaline phosphatase (ALP) secreted by osteoblasts catalyzes hydrolysis of ATP and ADP and therefore decreases the concentration of extracellular iPP and increases iP, enhancing bioavailability of ions for mineralization [41]. During the mineralization process, phosphate and calcium precipitate on the organic bone matrix forming hydroxyapatite crystals.

Calcitriol, 1,25dihydroxy-vitamin D_3_, is necessary for intestinal absorption of calcium. Bone is a dynamic tissue undergoing constant remodeling throughout life. As a result of this feature of bone, several metals accumulate in bone.

In view of the above, the** aim** of the present review was to provide updated information on the bone tissue effects of different, potentially toxic metals found in the natural and occupational environments.

Because exposure to toxic doses of copper and zinc has not been found to have toxic effects on bone, these two metals were not included in the present review.

## 2. Metal Toxicity in Bone Tissue

### 2.1. Cadmium

Clinical trials have shown that cadmium poisoning causes renal failure and osteoporosis and increases fracture risk [48, 49]. Two mechanisms of action of Cd on bone tissue are currently proposed [50]: a direct mechanism involving the direct action of the metal on bone cells [51] and an indirect mechanism by which Cd would first induce renal failure, increasing calcium and phosphorus excretion, decreasing vitamin D synthesis, and hence also decreasing calcium absorption in the digestive tract [52–54].

*In vivo* studies in experimental animals have shown that chronic exposure to Cd decreases mineralization of vertebral bodies, altering their biomechanical properties and rendering them more susceptible to deformity and fracture [55]. It is also well documented that Cd decreases expression of markers of osteoblastic differentiation (Runx2, osteocalcin), of extracellular bone matrix proteins (type I collagen), and of enzymes involved in the mineralization process (alkaline phosphatase-ALP) [56], altering the bone formation and mineralization process. Other studies provide evidence that chronic exposure to Cd decreases bone volume and increases the percentage of tartrate resistant acid phosphatase (TRAP) positive cells in subchondral tibial bone [57, 58]; the increase in TRAP activity would be an indication that osteopenia is induced by the increase in resorption. The percentage of fatty bone marrow has also been found to increase, suggesting that Cd inhibits differentiation of mesenchymal cells to osteoblasts, stimulating adipogenesis. The same study showed that Cd exerts different effects on different types of bone (long bones vs. craniofacial bone) [58].

*In vitro* studies have shown that Cd increases RANKL expression [59, 60], TRAP activity, and formation of TRAP-positive cells in the presence of RANKL [53] and stimulates formation of osteoclasts in cocultures of osteoblasts and osteoclast precursor cells [60]. The aforementioned studies suggest that Cd may regulate osteoblast expression of RANKL and indirectly induce osteoclastogenesis via RANKL. In addition, Cd has been shown to induce osteoblast apoptosis, by disrupting the cytoskeleton [61], as well as DNA fragmentation, an increase in the number of micronuclei and nuclear bridges [62], and an increase in reactive oxygen species, by activating the p38 MAPK pathway and inhibiting the Erk1/2 pathway [63].

Reports of coexposure to Cd and Pb published in the literature have shown that exposure to Cd and Pb may increase the risk of hearing loss and renal damage [64] and may have additive-toxic effects on the testicle, liver, and kidneys [65].

A study by Chen et al. [66] in a Chinese population exposed to Cd and Pb through foods showed that coexposure to these metals could have an interactive effect on bone mineral density in Chinese women.

### 2.2. Nickel

With regard to nickel and its adverse effect on bone tissue,* in vitro* studies showed that high concentrations of Ni inhibit alkaline phosphatase activity and consequently inhibit bone mineralization [67]. Kanaji et al. [68], however, found that osteocytes in culture have a cytotoxic effect, inducing cell apoptosis.

There are no reports in the literature on the effects of nickel poisoning on human bone or in* in vivo* experimental models.

### 2.3. Mercury

Mercury poisoning has been studied in goldfish. Methylmercury has been shown to decrease calcemia, increase metallothionein synthesis, and decrease estrogen receptor expression, directly affecting the metabolism of scale bone cells [69]. In their study in the marine teleost (*Girella punctata*) exposed to methylmercury or inorganic mercury, Yachiguchi et al. [70] reported an increase in metallothioneins and a decrease in TRAP and ALP expression. Mercury would therefore inhibit the activity of both osteoclasts and osteoblasts.

To date, there is only one report in the literature on the effect of mercury on bone tissue of mammals. ABD El-Aziz et al. [71] showed that prenatal intoxication of experimental animals with methylmercury has negative effects on rat fetus development, delaying ossification and decreasing long bone length.

There are no reports in the literature on the effects of mercury poisoning on bone in humans.

### 2.4. Chromium

*In vivo* studies in rats performed by Sankaramanivel et al. [72] showed that Cr(VI) accumulates in the femur and generates a systemic decrease in ALP and TRAP, suggesting an impact on both bone formation and resorption. De Lucca et al. [73] found that intoxication with hexavalent chromium caused a decrease in body and mandibular growth and in tooth eruption in experimental animals.

*In vitro* studies have shown that Cr(VI) is uptaken by osteoblasts through membrane transporters and is rapidly reduced to Cr(III), causing an increase in reactive oxygen species, oxidative stress, and damage to DNA; in addition, both Cr(VI) and Cr(III) decrease ALP activity and mineralization by osteoblasts in cultures [74, 75]. An* in vitro* study by Andrews et al. [76] showed that Cr(VI) decreases osteoblast survival as well as the number of osteoclasts and resorption.

There are no reports in the literature on the effects of chromium poisoning on bone in humans.

### 2.5. Lead

Lead (Pb) is deposited in both soft (liver, kidneys, heart, brain, and muscle) and hard tissues (bone). The chief target for Pb is the bone matrix, due to its ability to substitute other divalent cations in the body, such as Ca^2+^, Mg^2+^, and Fe^2+^, and though to a lesser degree, monovalent cations like Na^+^. Lead has a high affinity for thiol groups in the active sites of some enzymes. Lead poisoning affects mineral metabolism of calcium and phosphorus by inhibiting kidney 1-*α*-hydroxylase enzyme, which is required for 1,25-dihydroxy-vitD_3_ synthesis. This results in hypocalcemia and hypophosphatemia because of the decrease in intestinal absorption of both of these minerals [29].

Due to its accumulative effect in the body, tissue concentration of lead varies throughout an individual's life. In adults, 90-95% of total body lead is stored in trabecular and cortical bone, whereas, in children, 70 to 95% accumulates in trabecular bone as a result of its high turnover rate [77]. During pregnancy [78], lactation, and menopause, the increase in blood levels of lead stimulates lead accumulation in other body organs [79]. Therefore, the concentration of lead in blood and soft tissue organs can vary throughout life according to the metabolic activity of bone tissue.

Clinical studies have shown that lead accumulated in the body has negative effects on bone, decreasing cortical width and bone density and increasing fracture risk [80]. Moreover, experimental studies by Carmouche et al. [81] on fracture healing in lead-exposed animals showed that lead poisoning delayed cartilage formation and maturation and, consequently, delayed fracture healing. As to endochondral ossification, it has been shown that Pb affects skeletal growth by causing a decrease in stature, axial skeleton growth, and chest circumference during childhood [82, 83].

*In vivo* studies have shown that Pb poisoning decreases bone mineral content and the mechanical properties of long bones [84] and mandibular bone [85]. In addition, it generates an imbalance in bone remodeling, causing an increase in both bone formation (increase in amino terminal propeptide of type I collagen –P1NP) and resorption (increase in carboxy terminal telopeptide of type I collagen CTX). The bone formation/mineralization and resorption processes are accelerated, resulting in the formation of poor quality bone [86].* In vitro* studies provide evidence that Pb inhibits osteoblastic activity by inhibiting the Wnt signaling pathway [87] and induces osteoblast apoptosis [88]. Activation of autophagy by Pb (degrading damaged organelles) may play a protective role in osteoblast apoptosis [89].

With regard to endochondral ossification,* in vivo *studies have shown lead exposure to inhibit growth plate development in long bones [90] and* in vitro *studies have shown that Pb inhibits the expression of markers of growth plate chondrocyte phenotype (suppression of alkaline phosphatase and both type II and type X collagen expression) [91].

### 2.6. Aluminum

Once absorbed by the body, aluminum is incorporated to the bone matrix and is uptaken by osteoclasts during the resorption process. It deposits on trabecular bone surfaces and on the surfaces of the vascular canals that permeate compact bone. It has also been found to deposit on periosteal and endosteal surfaces [32]

Clinical studies provide evidence that Al poisoning causes bone diseases such as renal osteodystrophy [92, 93], osteomalacia [94], and osteoporosis [95]. Other studies by Chappard et al. [96] in patients with exostosis (the most frequent benign bone tumor in children and adults) showed that Al^3+^ and Fe^3+^ can substitute Ca^2+^ in the hydroxyapatite crystals.

*In vivo* studies have shown Al deposition in bone to decrease Ca, Mg, and P levels, inhibiting the bone mineralization process [97]. In addition, Sun et al. [98] found Al to inhibit osteoblast differentiation (as shown by a decrease in Runx2 expression), bone formation (through a decrease in the expression of type I collagen and IGF-1), and the Wnt/*β*-catenin pathway (via an increase in caspase 3 activity and expression), leading to osteoblast apoptosis.

In addition, Al has been shown to deposit in rat cartilage disrupting its histologic structure and inhibiting growth, as a result of inhibition of cartilage stimulatory growth factors (TGF*β*-1 and BMP-2) and a decrease in chondrocyte activity and extracellular matrix protein synthesis (a decrease in the expression of type II collagen and an increase in serum levels of the C-terminal telopeptide of type II collagen) [99].

*In vitro* studies provide evidence that Al inhibits osteoblast differentiation by inhibiting the BMP-2/Smad [100], ERK/MAPK, and Wnt/*β*-catenin pathways, decreases TGF-*β*1 expression by inhibiting type I collagen [101], and decreases the expression of osteopontin, osteocalcin, and bone sialoprotein as well as the concentration of extracellular calcium, mineralized matrix nodules, and ALP activity, thus inhibiting the bone mineralization process [102].

Degeratu et al. [103] developed a biomimetic polymer that mimics bone mineralization in the absence of cells. The authors incubated the polymer in different concentrations of Al^3+^ and found that aluminum altered the growth of calcospherites, inhibiting the mineralization process.

### 2.7. Titanium

Grenon et al. [104] found that titanium diffuses into new formed bone around and implant. Interestingly, Wennerberg et al. [105] found no correlation between implant roughness and release of ions.

Studies published in the literature have reported the release of titanium nanoparticles from metallic implants and have evaluated the effect of these particles on bone using in vitro experimental models that mimic the clinical situation [106, 107].* In vitro* studies by Zhu et al. [108] showed that titanium inhibits osteoblastic differentiation. Other reports have found titanium to inhibit expression of osterix, type I collagen [106], osteopontin, and osteocalcin and to delay ALP expression [109].

In addition, titanium has been found to stimulate osteoclastogenesis [110] and osteoclast activity in the presence of BMP-2 and RANKL [111].

### 2.8. Iron

Iron overload and related bone pathologies have been reported. Iron toxicity is due to excessive ingestion or systemic diseases that trigger accumulation of iron in the body, as is the case of hemochromatosis and thalassemia [112–114]. Clinical studies performed by Marcucci et al. [115] showed that patients presenting the most severe form of thalassemia major often develop a metabolic bone disease, leading to an alteration in the RANK/RANKL/OPG system, an increase in osteoclast activity, and osteoblastic dysfunction.

*In vivo* studies have shown that accumulation of iron in the body causes oxidative stress by increasing expression of proosteoclastogenic cytokines such as TNF*α* and interleukin-6 [116] and by inducing the JNK, ERK, and NF-к*β* pathways [117] which stimulates osteoclast differentiation and subsequently bone resorption. Iron has also been shown to inhibit endochondral ossification [118] and to decrease ostoeblastogenesis via a decrease in plasma levels of P1NP, Runx2, and BGLAP [119]. In the bone marrow, iron causes toxicity to mesenchymal cells [120], increases NOX4 expression promoting mesenchymal cell differentiation into adipocytes, and increases expression of caspase 3 triggering apoptosis.

*In vitro* studies have shown that iron promotes osteoclast differentiation [121], inhibits osteoblast differentiation [122] and activity [123], and could lead to osteoblast apoptosis via caspase 3 [124]. Iron overload also decreases formation of mineralization nodes [125] and inhibits growth of hydroxyapatite crystals, altering their crystallinity [126].

### 2.9. Arsenic

Arsenic has the ability to accumulate in soft (liver, spleen, and the gastrointestinal tract) and hard tissues. It has been suggested that arsenic competes with the phosphate group in hydroxyapatite crystals, forming apatite arsenate (Ca (5) (AsO(4)) (3)OH) and probably other calcium arsenate crystals [127].

Clinical epidemiological trials have shown association between arsenic poisoning and Paget's disease [128], which is caused by an imbalance in bone remodeling generated by an initial increase in resorption followed by excessive bone formation, which leads to pain, increase in fracture rates, and bone deformities [129].

In addition, there are clinical reports of osteomyelitis and osteonecrosis of alveolar bone caused by the use of dental paste containing arsenic trioxide to devitalize inflamed pulp prior to tissue removal in endodontic treatment [130–133].

There are few reports describing the* in vivo* effect of arsenic on bone tissue. A study conducted by Aybar Odstrcil et al. [134] showed that intoxication with low concentrations of arsenic inhibited long bone-endochondral ossification in the rat, due to the increase in growth cartilage width, particularly in the hypertrophic zone. In addition, Hu et al. [135] showed that intoxication with arsenic trioxide* in vivo* decreased maturation of osteoclast precursors (by decreasing RANKL expression) and osteoclastic activity (by decreasing TRAP activity), altering the bone resorption process. The authors also found that* in vitro* exposure to arsenic decreases osteoblastogenesis (due to a decrease in Runx2 expression and the VCAM-I adherence molecule) and osteoblastic activity (due to a decrease in ALP activity), affecting the bone formation process.

The ERK signaling pathway is involved in the cell-bone matrix interactions and in the osteoblast differentiation process.* In vivo* studies have shown that arsenic decreases bone mineral density and increases ERK phosphorylation, altering bone cell differentiation [136].

Furthermore,* in vitro* studies have demonstrated that arsenic inhibits proliferation and induces apoptosis of bone marrow derived mesenchymal cells (BMSCs) [137], osteoblasts, and chondrocytes [138], it generates an increase in reactive oxygen species in osteoblasts [139], and it induces apoptosis.

## 3. Conclusions

In low concentrations, some metals have an anabolic effect on bone tissue, as is the case of zinc, copper, and nickel. They mostly serve as cofactors for enzymes involved in bone remodeling processes. However, both the lack and excess of these metals in the body can damage bone integrity. Whether their effect is beneficial or toxic depends on external (environmental concentration and nutrition) and internal factors (absorption and metabolism of these elements, genetic disposition, age, and gender) and on their mutual interactions [140]. Other metals, such as cadmium, arsenic, mercury, chromium, and aluminum are toxic to bone cells even at low concentrations.

As shown in the present review, most of the metals analyzed here are found in nature in low levels. However, human activities are the major cause of pollution, and exposure to these trace elements causes severe health problems. The present review focused on their effect on bone.

Bone tissue undergoes constant remodeling throughout life. The process involves the coordinated action of resorption, synthesis, and mineralization of the bone matrix. Overall, metals pose two problems: on one hand, their direct toxicity on bone cells and, on the other, their accumulation in the bone matrix. As shown here, their direct toxicity mainly affects osteoblasts, inhibiting osteoblast differentiation, synthesis activity, and mineralization of the extracellular matrix. Their effect on osteoclasts differs according to the metal, increasing or decreasing TRAP enzyme activity and inhibiting maturation of precursors. This generates an imbalance in the bone remodeling process, decreasing bone formation and contributing to the generation of bone diseases such as osteopenia and osteoporosis. In addition, the ability of trace metals to accumulate in the extracellular bone matrix allows bioaccumulation and thus leads to an increase in the half-life of the metal in the body. This is of particular importance when exposure levels are low but exposure is constant over time, since, in the long term, the deleterious effects could be the same or worse than short exposure to high levels of the metal.

**Figure 1:**
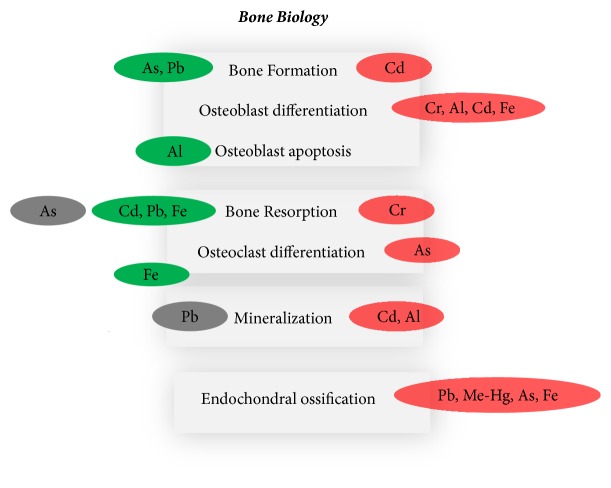
Schematic diagram showing the main* in vivo *effects of metals on bone. Increase or stimulation (red), decrease or inhibition (green), alteration (increase or decrease) (grey).

Figure 1 shows a schematic diagram of the main* in vivo *effects of metals on bone.

Despite the many advances in our knowledge of the effect of metals on bone tissue, much remains to be elucidated. Understanding their mechanism of action will enable finding adequate treatments to counteract their negative impact on bone.

## Figures and Tables

**Table 1:** 

Element	Levels	References
Cadmium	< 1*μ*g/L in urine	[42]
Zinc	no records	none
Copper	70-140 *μ*g/dL in plasma	[43]
Nickel	1-3 *μ*g/L in urine 0.2 *μ*g/L in plasma	[44]
Mercury	< 7*μ*g/L in urine < 5*μ*g/L in blood	[42]
Chromium	<0.2 *μ*g/L in urine 0.7-28 *μ*g/L in blood 0.1-0.2 *μ*g/L in plasma	[43]
Lead	100 *μ*g/L in blood Not detectable in urine	[45]
Aluminum	1-3 *μ*g/L in plasma	[46]
Titanium	no records	none
Iron	no records	none
Arsenic	1 *μ*g/L in blood	[47]
